# Commercial milk formula marketing following increased restrictions in Singapore: A qualitative study

**DOI:** 10.1111/mcn.13562

**Published:** 2023-09-05

**Authors:** Chompoonut Topothai, Grace Ping Ping Tan, Yvette van der Eijk

**Affiliations:** ^1^ Saw Swee Hock School of Public Health National University of Singapore Singapore Singapore; ^2^ International Health Policy Programme Ministry of Public Health Nonthaburi Thailand

**Keywords:** breastfeeding, commercial milk formula, International Code of Marketing of Breast‐milk Substitutes, marketing, milk substitutes, promotion, Singapore

## Abstract

The promotion of commercial milk formula (CMF) negatively impacts breastfeeding outcomes. In 2019, Singapore updated its 1979 Code of Ethics of the Sale of Infant Foods Ethics Committee Singapore (SIFECS) to increase marketing restrictions on CMF for infants 0–12 months. However, little is known about industry tactics to undermine these restrictions. This qualitative study explores health workers' and mothers' experiences with CMF marketing in Singapore following the 2019 restrictions. We conducted a qualitative study, using semistructured interviews with 14 mothers of infants aged less than 5 months and 20 health workers with expertise in antenatal, maternity, or paediatric care. We analysed data thematically using inductive coding. Five themes were identified. Mothers and health workers reported digital marketing, product line extensions with toddlers' milk and milk for mothers, and CMF sponsorships in the healthcare setting. Expert endorsement, competitive price, nutritional claims, and brand reputation influenced mothers' infant formula choices, yet both mothers and health workers appeared to be unaware of the impact of CMF marketing tactics on their own perceptions. The restriction of CMF marketing and infant feeding practices varied widely between hospitals, with private hospitals and practices having less strict controls on CMF marketing. Despite the updated SIFECS restrictions, CMF companies continue to target mothers and health workers in Singapore. SIFECS restrictions should be tightened to align with international guidelines, by increasing their scope to include toddlers' milk and prohibiting cross‐promotion, digital marketing, and any sponsorships of events targeting health workers that may create a conflict of interest.

## Key message

- Mothers' and health professionals' exposure to commercial milk formula (CMF) promotion was still widespread in Singapore even after the new CMF restriction was implemented in 2019.
- Digital marketing, distribution of free samples of milk for toddlers and mothers, dubious premium health and nutrition claims, and sponsorship from CMF industries were common tactics found in this study.
- Updating CMF restrictions to align with the Code, including restricting CMF marketed for toddler milk, prohibiting cross‐promotion, and disallowing sponsorship from CMF industries, together with an effective enforcement system, may facilitate better control of unethical CMF promotions in Singapore.

## Graphical abstract

Despite the updated restrictions, commercial milk formula companies continue to target mothers and health workers in Singapore. Restrictions should be tightened to include toddlers' milk and prohibit cross‐promotion, digital marketing, and any sponsorships of events targeting health workers that may create a conflict of interest.

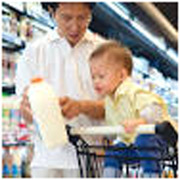

## 1 INTRODUCTION

According to World Health Organization (WHO) and UNICEF guidelines, infants should be exclusively breastfed in the first 6 months and continually breastfed with appropriate complementary feeding until at least age 2 years (World Health Organization, & UNICEF, 2003), yet less than half of the infants globally are breastfed optimally (World Health Organization, 2022a). While mothers' decisions on infant feeding are influenced by multiple factors (Baker et al., 2023; Pérez‐Escamilla et al., 2023), a well‐established body of literature shows that the promotion of commercial milk formula (CMF), which refers to formulas for children aged 0–36 months, negatively impacts women's attitudes to breastfeeding (Piwoz & Huffman, 2015; Rollins et al., 2016, 2023; World Health Organization, 2022b) and encourages early termination of breastfeeding (Lisi et al., 2021; Romo‐Palafox et al., 2020; Sadacharan et al., 2013).

To address this issue, WHO and its member states adopted the International Code of Marketing of Breast‐milk Substitutes in 1981 (World Health Assembly, 1981) and subsequent relevant resolutions (together referred to as ‘the Code’) (World Health Organization, 2022c). The Code restricts CMF promotions to the public and in healthcare settings to enable parents to make more informed choices on infant feeding. The Code complements other breastfeeding promotion interventions (Robinson et al., 2019), especially the Baby‐Friendly Hospital Initiative (BFHI) which sets standard practices to promote breastfeeding and prohibits all forms of CMF promotion in healthcare settings (Robinson et al., 2019; UNICEF, 2023; World Health Organization, 2017b, 2022c).

Singapore, a high‐income country in Southeast Asia, faces typical challenges in balancing CMF marketing practices with breastfeeding promotion (Baker et al., 2023; Berry et al., 2012). Efforts to limit CMF marketing are based on the Code of Ethics of the Sale of Infant Foods Ethics Committee Singapore (SIFECS), first formulated in 1979 and updated with tighter restrictions in 2019 (Health Promotion Board, 2019; Health Promotion Board Singapore, 2019). In 2019, SIFECS prohibited the marketing of CMF for infants aged 0–12 months and prohibited free sample distributions and sponsorships in healthcare institutions (Health Promotion Board, 2019). Although SIFECS is a voluntary Code, the violators receive warning letters while repeat violations are publicised on a government website (Health Promotion Board Singapore, 2019). Thus Singapore does restrict CMF marketing but not to the extent to which it would be aligned with the Code that covers CMF products from 0 to 36 months, bans cross‐promotion, and prohibits sponsorship of events targeted health workers (World Health Organization, 2022c).

While breastfeeding initiation in Singapore was found to be near‐universal at 99%, exclusive breastfeeding (EBF) rates were still low, just 28% at 2 months (Chua & Win, 2013). In 2019, the exclusive breastfeeding rate at 6 months was 38% (De Roza et al., 2019). The literature on breastfeeding in Singapore has explored experiences and difficulties (Jia Choo & Ryan, 2015), attitudes and determinants (Lau et al., 2017), and factors associated with EBF (De Roza et al., 2019; Su et al., 2007). However, little is known about how CMF is marketed and its potential influence on breastfeeding practices, especially in light of the 2019 SIFECS implementation. Thus, this study aimed to explore CMF marketing in Singapore following the 2019 SIFECS restriction from the perspectives of health workers and new mothers.

## 2 METHODS

### 2.1 Reflexivity

We, the research team, were composed of three female members from the Saw Swee Hock School of Public Health, National University of Singapore. C. T. is a doctor who is currently enroled in the Ph.D. programme, G. T. is a research associate, and Y. V. is an assistant professor with Ph.D. credentials. Each researcher has prior experience in conducting qualitative studies. All of us have experience in breastfeeding our child, prior knowledge, and personal interest in infant feeding topics and Code. C. T. has additional expertise in Code implementation.

### 2.2 Study design

Y. V. conceptualised and designed the study. We used a single‐method qualitative study design, drawing from semistructured interviews with 14 mothers and 20 health workers. Our research question was the following: ‘What is the current practice of CMF marketing and how does it affect knowledge, perceptions, attitudes, and practices related to breastfeeding and formula feeding among Singaporean mothers and healthcare workers?’ We applied an interpretative approach to this study as it acknowledges the significance of context in shaping individuals' perspectives and values the individual's subjectivity. Thus, this approach allowed us to understand the rich diversity of participants' experiences through the interpretation of their viewpoints embedded in particular contexts which could be influenced by various social, cultural, and historical backgrounds (Starks & Brown Trinidad, 2007).

### 2.3 Sampling and recruitment

We used purposive sampling to identify mothers with a range of sociodemographics, the type of hospital used, and infant feeding patterns. We recruited mothers via flyers shared on the social media of local mothers' support groups, authors' professional networks, and snowball recruitment. Mothers who were interested in participating subsequently registered on the Google form and received an invitation to participate. All mothers were Singaporean citizens or Permanent residents who had used obstetric‐gynaecological (ob‐gyn), maternity, and/or paediatric services in Singapore within 8 months before an interview and had at least one infant below the age of 5 months.

We used purposive sampling and snowballing techniques to recruit health workers, with diversity by area of expertise related to infant feeding. We first approached health professionals who were well‐known for breastfeeding promotion in Singapore, both publicly and within our professional network, and then snowballed from them to recruit other participants. G. T. recruited all health workers via email invitations. All health workers had expertise working in general medicine, maternity, obstetrics‐gynaecologist (ob‐gynes), and/or paediatric care in public or private healthcare settings in Singapore (Table 1).

**Table 1:** Participant characteristics.

	Total number
*Mothers*	
Age (years)	
27–30	4
31–33	4
34–36	6
Ethnicity	
Chinese	12
Other (Vietnamese, Indian)	2
Monthly household income (Singapore Dollars)	
$6000 or less	1
>$6000–$10,000	4
>$10,000–$14,000	5
More than $14,000	4
Number of children	
1	7
2	5
3	2
Type of hospital (most recent delivery)	
Public	8
Private	6
Hospital implemented Baby Friendly Hospital Initiative (most recent delivery)	
Yes	10
No	4
Breastfeeding or formula feeding (youngest child)	
Exclusively breastfeeding (EBF)	7
Not exclusively breastfeeding (non‐EBF)	7
*Health workers*	
Role	
Doctor (women's health)—obstetrics‐gynecology or perinatal psychiatry	5
Doctor (paediatric)	3
General practitioner—polyclinic nurse or doctor	2
Nurse—with experience in maternity or obstetric‐gynecology care	5
Lactation consultant	3
Researcher—with a background in child nutrition	2

### 2.4 Data collection

G. T. and Y. V. developed an interview guide based on the literature related to formula feeding, CMF marketing, and the prohibitions indicated in the SIFECS (The Sale of Infant Foods Ethics Committee of Singapore, 2020). The interview guide is presented as a Supporting Information file. We obtained written informed consent before the interview by informing participants of the study goals, procedures, risks, and confidentiality. G. T. conducted all semistructured interviews from June 2021 to May 2022, using Zoom conferencing software, in English—the most widely spoken language in Singapore. Interviews lasted 45–90 min each and were audio recorded, and transcribed verbatim. We reimbursed each interviewee with S$20 cash.

### 2.5 Analysis

C. T. and G. T. independently coded the transcripts line‐by‐line in NVivo (version 14) using an inductive approach. Based on the verbatim, we developed an initial codebook with inductive codes, after which each transcript was independently double‐coded and compared to ensure coding consistency. Similar codes were combined into new codes and were added to the codebook during the coding process. Discrepancies were reviewed and discussed until a consensus was reached in an iterative process with all three authors. Codes were then organised into categories, subcategories, and overarching themes. See the Supporting Information file for the codebook.

### 2.6 Ethics approval and consent to participate

Ethics approval was obtained from the University's Institutional Review Board. All human participants' research was performed in accordance with relevant guidelines and regulations. All participants received an extensive briefing of the study and provided informed consent before participation.

## 3 RESULTS

The mothers' median age was 32 years old (27–36) years old. Most mothers were Chinese and had a monthly household income of more than 10,000 Singapore dollars. Half of the mothers had a single child and practiced exclusive breastfeeding. More than half of mothers received the most recent health services from public hospitals, which have been certified with BFHI. Health workers included nurses working in ob‐gynes and maternity settings, doctors specialising in women's care, paediatricians, general care practitioners, lactation consultants, and researchers specialising in infant nutrition.

We identified five themes: (1) marketing to the general public, (2) marketing in the healthcare setting, (3) health workers' attitudes towards marketing and its restriction, (4) health workers' attitudes and practices on infant feeding, and (5) mothers' attitudes towards marketing and formula feeding.

### 3.1 Marketing to the general public

#### 3.1.1 As experienced by mothers

Mothers described their experiences of being heavily targeted by online CMF promotions, with targeted advertising on social media and search engines, and thorough follow‐up via email and telephone from CMF industry representatives after subscribing to their mailing lists:

I signed up for some of the samples … So after that, they would routinely call me to offer samples for the next stage.

—Mother (EBF, private hospital)

Mothers also recalled receiving free samples and discount coupons for CMF products and these promotions encourage brand popularity and loyalty:

I remember when my child was almost two (months) and we switched to Aptamil because they gave you vouchers, and it is cheaper… That's how they made their market in Singapore. So Aptamil is now very popular.

—Mother (EBF, private hospital)

#### 3.1.2 As observed by health workers

Health professionals shared their observations of CMF marketing, however, in a broader way than mothers. They noticed how CMF companies promote products to parents with attractive free gifts and by disseminating educational materials with research data, using premium nutrition claims that their products have unique properties to other brands, and using line extensions to cross‐promote products that share similar branding and packaging:

They (CMF companies) actually start you (mothers) from when you are pregnant, maternal milk… Then they also move on to toddler milk… They (CMF companies) have this special formula for picky eaters.

—Lactation consultant, public hospital

### 3.2 Marketing in the healthcare setting

#### 3.2.1 Marketing targeting mothers

Mothers described encountering CMF logos and brand names in a range of places, including on items such as growth charts and toys displayed in maternal and child health clinics and formula milk displayed in special booths at hospital pharmacies. Some mothers had received free CMF samples without request, mostly from paediatric and ob‐gyn practices, in the form of toddler milk and milk for pregnant women or postpartum mothers. While toddler milk is not for infants aged 0–12 months, they were marketed with the same branding. Hospital discharge bags given to mothers also contained promotional materials, such as coupons and gifts; however, none of them provided infant formula samples:

[In the private hospital] there are two kinds of discharge bags. One is prepared by [the hospital], another one, I think is another bag that is by a formula brand… you will see Friso giving you bottles, containers, all that, with their names… Similac also gave out some bibs.

—Mother (EBF, public hospital)

Mothers generally noted that hospitals prohibit promotions of CMF for infants aged 0–12 months and encourage breastfeeding. One mother noticed that the hospital rotated the infant formula milk brands used in the hospital:

Even if you (mothers) contact them (CMF companies) when the kid is born, they wouldn't offer you any samples until after six months. So they cannot offer any samples for stage one I think, it's against the local guidelines.

—Mother (EBF, private hospital)

#### 3.2.2 Marketing targeting health workers

Ob‐gyn doctors, paediatricians, and lactation consultants revealed that CMF industry representatives still visit them to give information about formula products and provide free gifts and samples of toddlers' or mothers' milk, with some requesting their endorsement to promote such products to mothers. It was observed that CMF industries increasingly promoted mothers' milk through healthcare settings:

They say, ‘Would you like to take some of this milk for the mother?’ They had done their own study that it helps the mother, those who are not eating well. Sometimes we do take some mother's milk… We put it out there, indirectly in a way, telling moms, ‘If on that day you're too busy, you're unable to eat well…then you have a drink because it's a pack of goodness.’

—Lactation consultant, private practice

#### 3.2.3 Seeking partnership with health workers

While some health workers were unaware of collaborations with the CMF industry, others described CMF industry efforts to build partnerships with health professionals, especially paediatricians, via sponsored educational talks, overseas conferences, and research collaborations:

…they don't pedal milk to you, but they will want to collaborate with the College of [Paediatricians], with professional societies to say, ‘Hey, let's sponsor some talks. We can organise this and that.’ I think those are subtle ways that they are still trying to maintain the relationship.

—Paediatrician, public hospital

…we have Journal Club, then they tried to sponsor the food for Journal Club and the [industry] guys are educating us about new developments in formula milk.

—Women's health doctor, public hospital

### 3.3 Health workers' attitudes towards marketing and its restriction

#### 3.3.1 Views on the industry and its marketing

Health workers recognised that CMF companies are profit‐based, well‐resourced organisations capable of targeting parents with attractive marketing, but they had diverse views towards CMF marketing. Some believed that CMF marketing has been sufficiently regulated or is not a problem in Singapore, while others believed it still creates misconceptions about infant feeding through sophisticated marketing tactics, calling for tighter restrictions:

[Marketing] for mother's milk, I think, it's no harm. It's a form of beverage. I think that should be okay. You're helping moms if they're unable to eat well…like the essence of chicken.

—Lactation consultant, private practice

Even physicians as well, they get the misconception that there's nothing wrong with formula feeding, it's as good as breastfeeding. We have spoken to moms as well who actually think that formula is superior to breast milk.

—Paediatrician, public hospital

#### 3.3.2 Views on industry partnership

Health workers also had mixed views on partnering with the CMF industry on research. While some highlighted the practical value of partnering with CMF companies, others believed that such partnerships should be handled with caution due to the industry's vested interests. Others believed that partnerships with milk industries should be prohibited:

…funding for research is competitive and you have to be realistic… They have an influence on the world. They're also stakeholders… When their companies were founded, they were solving problems. Things changed a lot, of course, so we have to all pivot to the new norms.

—Paediatrician, public hospital

The research is always a bit discounted because definitely, we can't exclude some covert influence. Sometimes you may not even realise that you've been influenced.

—Women's health doctor, public hospital

#### 3.3.3 Awareness of and adherence to restrictions

Healthcare professionals were generally aware that CMF marketing is strictly regulated in healthcare settings; however, their awareness of the revised SIFECS measures varied depending on their area of expertise and involvement in the regulation process. Some who were unaware of SIFECS still recognised that partnerships with CMF companies are discouraged. Participants with research experience proposed that ethical policies related to research sponsored by CMF industries should be established to regulate collaboration between the health sector and CMF industries:

I don't think we (the hospital) have a blanket policy, per se, but I don't think we (health workers) are encouraged to do research or collaborate with the formula milk industry… given that we are 100% breastfeeding hospital.

—Women's health doctor, public hospital

Healthcare professionals who were aware of SIFECS noted that all hospitals in Singapore must abide by its measures. Some participants reported their hospitals to have concrete policies that ban all infant formula promotions and brand endorsement, such as discontinuing the sale of infant formula, rejecting sponsorship from the CMF industries, and forbidding the display of CMF logos. Two participants from public healthcare settings also mentioned having a focal point in their organisations to deal with CMF industry representatives as part of the SIFECS implementation:

I would say that, at least I can speak for my hospital, for anything to deal with formula, they (hospital staff) come to me. Even our corporate communications, they know and they're very mindful.

—Paediatrician, public hospital

Healthcare professionals described a considerable variation in CMF marketing restrictions between private and public healthcare settings, with promotions being more common in private settings where the regulation is less strict:

…in the private hospitals, as far as I know, that's where most of these formula milk companies make their money. I think they're not as strict (as public hospitals), and they're clearly more pro‐choice, they run it more like a service up there, compared to an institution.

—Women's health doctor, public hospital

### 3.4 Health workers' attitudes and practices on infant feeding

#### 3.4.1 Views on formula feeding

Most healthcare professionals hold that formula feeding is necessary and appropriate in some circumstances, notably when medically indicated or when breastfeeding cannot be established. Some also considered CMF as an appropriate supplement for mothers unable to produce enough breast milk. However, two health professionals believed that CMF could be more nutritious than breast milk in some cases:

If I'm not wrong, there are some pieces of evidence that say that formula milk is actually more nutritious than breast milk after the first six months.

—Women's health doctor, public hospital

A smaller number of health workers expressed concerns regarding the widespread use of CMF without proper indication, while one suggested classifying CMF as a medicine that requires a prescription to ensure its appropriate usage:

It used to be a solution, and now it's become a problem. In our world, half a century ago, we needed infant formula because the mothers were undernourished.

—Paediatrician, public hospital

I think that's what we are aiming for, only special kids… they (special kids) have to have a specially designed formula, then they will have to be given by their paediatrician. They (CMF products) shouldn't actually be available for selling.

—General practitioner, polyclinic

#### 3.4.2 Factors influencing feeding advice

When asked how they approach cases of mothers who opt to formula feed without medical indication, most health workers indicated that they would first explore the mother's underlying reasons for choosing CMF and address any underlying misconceptions before giving any further advice. However, they emphasised the need to respect the patient's choice after the informed decision is made:

I think it's still a personal choice, but I believe that it is important for them to make an informed decision and at the same time, not to make them feel like they are any less of a parent just because they choose to give formula.

—Paediatrician, public hospital

Some also cited personal interest, age, and gender as factors that influenced the feeding advice given by health workers. Younger doctors were observed to be more probreastfeeding than older doctors due to a stronger emphasis on nutrition in their education curriculums, and female doctors were described as being more personally invested in breastfeeding issues than male doctors.

#### 3.4.3 Mothers' experiences with feeding advice

When asked about the infant feeding advice given to them when engaging in antenatal, maternity, and paediatric care, most mothers generally described the advice as being probreastfeeding. Breastfeeding was heavily promoted through various channels in the healthcare setting, such as direct advice from doctors and nurses, pamphlets, and parenting courses. A few mothers even felt that breastfeeding promotion was too strong as if it was the only option they had. However, two breastfeeding mothers reported being asked about their feeding choices as if breastfeeding was optional, and many mothers reported being offered formula by health professionals during their hospital stay which sent a mixed message:

Before I gave birth they already asked, ‘Do you intend to breastfeed, or do you intend to feed formula?’… They make it sound as if breastfeeding is optional.

—Mother (EBF, public hospital)

### 3.5 Mothers' attitudes towards marketing and formula feeding

#### 3.5.1 Mothers' formula brand choices

Marketing appeared to influence mothers' CMF brand choice, regardless of breastfeeding experience. Expert endorsement, particularly by paediatricians, was the most cited reason for brand choice:

My baby had some colic issues… PD [paediatrician] recommend[ed] Similac… That's why we decided to change to Similac… I'm not sure why the PD chose that brand.

—Mother (Non‐EBF, public hospital)

Furthermore, mothers generally believed that high quality was conveyed by price, so they tended to choose more expensive brands, although some mothers preferred the cheaper value brands. Nutritional components were another criterion considered important by some mothers. A number of mothers believed there were no major differences between brands, yet some felt that established brands gave higher assurance of product quality and safety:

I should just pick the most expensive one because I already could not give my kid enough breast milk, so I thought I'd better pick the most expensive one, assuming that that would be the best.

—Mother (EBF, private hospital)

What is the company? Is it Abbott? Is it Nestlé? I would see whether it is something that is established, basically, so that at least it's not so scary.

—Mother (Non‐EBF, public hospital)

#### 3.5.2 Views on marketing

Two breastfeeding mothers felt that CMF marketing was relatively innocuous and should not probably undermine breastfeeding practice:

There's a lot of hoo‐ha over the formula companies advertising but to be honest, for me, I don't think it's a huge factor… everyone does know that breast milk is best. It's not because they've been brainwashed by the formula companies. It's just personal constraints that make breastfeeding very difficult.

—Mother (EBF, public hospital)

However, some mothers felt that the nutritional claims of CMF products were dubious and misleading for consumers:

Pediasure is a big thing because they always talk about picky eaters. Picky eaters, if you give them formula milk, that's not going to help with picky eating.

—Mother (EBF, private hospital)

#### 3.5.3 Views on formula feeding

Exclusively breastfeeding mothers viewed formula feeding as unnecessary and unhealthy due to the addition of artificial contents such as sugar and additives, whereas formula‐feeding mothers believed there was no difference between formula milk and breast milk. Despite variations in feeding practices, mothers generally considered being fed as the most crucial issue rather than the type of milk. Mothers also felt that formula feeding is a matter of personal choice, shaped by a range of factors:

I think some parents tend to overdo it to the extent that they think that formula is poison. This is also quite bad because sometimes it comes to a point whereby they neglect whether their child is fully fed or not.

—Mother (EBF, public hospital)

Some people, choose to 100% feed their child formula and the child also grows up healthy and happy. I don't think it is a negative thing, it is just the decision of the parents how to feed your child.”

—Mother (Non‐EBF, public hospital)

## 4 DISCUSSION

To our knowledge, this is the first study exploring CMF marketing practices after the 2019 SIFECS amendment in Singapore. With tighter CMF marketing restrictions, the promotion of CMF for infants aged 0–12 months appeared to be prohibited as it was not reported in this study. However, mothers and healthcare professionals continue to be exposed to CMF marketing online and in healthcare settings, particularly through targeted digital advertising, promotions, free sample distribution of toddlers' and mothers' milk, claims of nutritional benefits, and sponsorships in a bid to forge partnerships with healthcare professionals. These findings are consistent with previous studies that reported similar marketing used by CMF industries worldwide (Becker, Zambrano, et al., 2022; Berry et al., 2012; Cattaneo et al., 2015; Han et al., 2022; Hernández‐Cordero et al., 2019; Hidayana et al., 2017; Jones et al., 2022; Liu et al., 2014; Merewood et al., 2010; Nguyen et al., 2021; Parrilla‐Rodríguez & Gorrín‐Peralta, 2008; Pereira‐Kotze et al., 2022; Sheikh et al., 2022; Vinje et al., 2017), especially the sharp increase in digital marketing for CMF since the COVID‐19 pandemic (Ching et al., 2021).

CMF marketing applied the same playbooks used elsewhere by targeting pregnant women, new mothers, and healthcare workers (Chen et al., 2015; World Health Organization, 2022b; Wright, 2006). Some participants appeared to be unaware of such targeted marketing and its potential influence on their attitudes and practices. Some mothers still regarded CMF marketing as relatively innocuous, despite our finding that mothers' formula brand choices were based on various marketing components, including the price which conveys premium quality, nutritional claims, trusted or well‐established brand, or experts' endorsement, consistent with findings in other countries (Romo‐Palafox et al., 2020; World Health Organization, 2022b). The distribution of mothers' milk in health facilities is concerning, as it may reinforce the perception that CMF is essential along with cross‐promoting the CMF for infants, leading to greater CMF usage in the future. Thus it is important for the government to provide more accurate information and public communication about CMF products, including those CMF line extensions targeted to mothers.

Some health workers still viewed CMF marketing as relatively benign and welcomed research partnerships with CMF companies, despite evidence that consistently shows that these practices influence the feeding advice given to mothers, leading to negative breastfeeding outcomes (Doherty et al., 2022; Hernandez‐Cordero et al., 2022; Piwoz & Huffman, 2015; Rosenberg et al., 2008; Rothstein et al., 2021; Sobel et al., 2011). Health workers may hold the belief that partnering with the CMF industries does not create a conflict of interest as long as their professional judgement is not compromised, or may prioritise the benefits of collaboration as sponsorships for research collaborations are the most common offer for health professionals by CMF industries (Genevieve Ellen et al., 2022).

Therefore, our findings showed that the current SIFECS was insufficient in preventing CMF companies from actively infiltrating the health system through sponsorship. Similar tactics were reported elsewhere (Baker et al., 2021; Laurence et al., 2019). This highlights a need for responsible agencies, such as professional committees, academic institutes, government, and hospital leaders, to provide sufficient continued professional development to health workers, such as skill development to support infant feeding, and set the standard ethical practice by discouraging sponsorships from CMF companies to avoid conflicts of interest (Becker, Ching, et al., 2022; Rollins et al., 2023). It also underscores the importance of raising awareness among health workers on the impact of CMF marketing and the potential impacts of conflicts of interest with the CMF industry in patient care (Wright, 2006).

This study shows that the increased restriction of SIFECS appears to have limited success in eliminating CMF marketing in Singapore. This unsuccessful restriction could be due to SIFECS loopholes that ban only the promotion of CMF for infants aged 0–12 months, enabling the marketing through product line extension in healthcare settings and other online platforms (Berry et al., 2012; Jones et al., 2022; Karageuzián et al., 2021). Thus, SIFECS should be further strengthened with an aim to align with the Code, which prohibits the marketing of CMF for children aged 0–36 months and cross‐promotion (World Health Organization, 2017a). Online marketing channels should be closely monitored since Singaporeans have a high rate of internet access and use (Sue Howe, 2023). Future studies should document the frequency, and profile of promotion that may violate SIFECS by specific CMF industries and publicise them on government websites.

Another reason for the unsuccessful CMF restriction could be the lack of implementation of SIFECS, as some health professionals were unaware of the SIFECS policy. Our study highlights the importance of communication and training on SIFECS for health professionals, especially those working in antenatal, maternity, or paediatric settings. The seamless integration of SIFECS as part of BFHI implementation would encourage better SIFECS implementation. In Vietnam, for instance, Code violations found in health facilities are counted as a penalty criterion giving a lower score for the BFHI reaccreditation system (Nguyen et al., 2021). The reinforcement of following the 10 steps to successful breastfeeding among health workers would also enhance the compliance of SIFECS since health workers may be aware of the necessity of regulating CMF marketing in health facilities to prevent confusing messages on infant feeding when encouraging mothers to breastfeed. Additionally, the government should make efforts to ensure that the remaining maternal facilities in Singapore receive BFHI verification, especially private hospitals that were found to have fewer restrictions on CMF marketing, as the literature showed that healthcare settings that complied with BFHI were more likely to have better breastfeeding outcomes (Pérez‐Escamilla et al., 2016; Shing et al., 2022).

While our findings illustrate CMF marketing practices in the Singapore context, it present similar trends faced by other neighbouring countries, for instance, highly prevalent CMF digital marketing and advertisement targeted mothers in Thailand (Cetthakrikul et al., 2022) and Vietnam (Nguyen et al., 2021), engagement between health professionals and CMF companies through sponsorship in Philippines (Baker et al., 2021), aggressive marketing of growing‐up milk and CMF for mothers and line extension using the design of similar pictures, logos, or packaging to cross‐promoted to CMF for infants in many South‐East Asia countries (Vinje et al., 2017), and the use of marketing content focusing on premiumization and nutrition benefits in China (Han et al., 2022). Our findings even mirror the situation found in other high‐income countries, especially the marketing pattern of toddler milk in the United States (Richter et al., 2022; Romo‐Palafox et al., 2020). From the marketing pattern shared across the region, our evidence underscores that the voluntary Code cannot regulate unethical CMF marketing practices (Piwoz & Huffman, 2015; Rollins et al., 2016). Furthermore, even countries with legislative measures but no strong enforcement systems could not regulate aggressive CMF marketing practices (Lutter, 2013; Sokol et al., 2008). Singapore may well see marked improvements in exclusive breastfeeding practices by legislating the voluntary SIFECS to be a legally binding instrument with strong regulatory enforcement.

### 4.1 Limitations

Although we provided critical reflection, and reported on the research process and deviant cases, to ensure the credibility of the research, our study has limitations. It is a qualitative study exploring CMF marketing from the perspectives of mothers and health workers following the updated SIFECS restrictions; findings may not be transferrable to other contexts, and measurement of the impact of SIFECS on healthcare practices would require a pre‐post longitudinal design. Social desirability bias could occur when health workers discuss their workplace's policy, but we triangulated this information with mothers' experiences to reduce the effect of this potential bias.

## 5 CONCLUSION

Despite updated CMF marketing restrictions, mothers and healthcare professionals in Singapore continue to be exposed to CMF marketing in the healthcare setting and online, and such marketing appears to influence infant feeding attitudes and practices. While the 5th edition of SIFECS being implemented in 2019 could partially restrict some CMF marketing in healthcare settings, its effectiveness could be enhanced if its provisions were strengthened to be aligned with the Code, with effective enforcement mechanisms.

## AUTHOR CONTRIBUTIONS

Chompoonut Topothai, Grace Ping Ping Tan, and Yvette van der Eijk performed the research. Yvette van der Eijk designed the research study. Grace Ping Ping Tan conducted all interviews. Chompoonut Topothai and Grace Ping Ping Tan analysed the data. Chompoonut Topothai wrote the manuscript. All authors reviewed and approved the final draft before submission.

## CONFLICT OF INTEREST STATEMENT

The authors declare no conflict of interest.

## Supporting information

Supporting information.

Supporting information.

## Data Availability

Data that support the findings of this study are available in the supplementary material of this article.

## References

[mcn13562-bib-0001] Baker, P. , Russ, K. , Kang, M. , Santos, T. M. , Neves, P. A. R. , Smith, J. , Kingston, G. , Mialon, M. , Lawrence, M. , Wood, B. , Moodie, R. , Clark, D. , Sievert, K. , Boatwright, M. , & McCoy, D. (2021). Globalization, first‐foods systems transformations and corporate power: A synthesis of literature and data on the market and political practices of the transnational baby food industry. Globalization and Health, 17(1), 58. 10.1186/s12992-021-00708-1 34020657 PMC8139375

[mcn13562-bib-0002] Baker, P. , Smith, J. P. , Garde, A. , Grummer‐Strawn, L. M. , Wood, B. , Sen, G. , Hastings, G. , Pérez‐Escamilla, R. , Ling, C. Y. , Rollins, N. , & McCoy, D. (2023). The political economy of infant and young child feeding: Confronting corporate power, overcoming structural barriers, and accelerating progress. The Lancet, 401(10375), 503–524. 10.1016/S0140-6736(22)01933-X 36764315

[mcn13562-bib-0003] Becker, G. E. , Ching, C. , Nguyen, T. T. , Cashin, J. , Zambrano, P. , & Mathisen, R. (2022). Babies before business: Protecting the integrity of health professionals from institutional conflict of interest. BMJ Global Health, 7(8), e009640. 10.1136/bmjgh-2022-009640 PMC9358938 35926917

[mcn13562-bib-0004] Becker, G. E. , Zambrano, P. , Ching, C. , Cashin, J. , Burns, A. , Policarpo, E. , Datu‐Sanguyo, J. , & Mathisen, R. (2022). Global evidence of persistent violations of the international code of marketing of breast‐milk substitutes: A systematic scoping review. Maternal & Child Nutrition, 18 (Suppl. 3), 13335. 10.1111/mcn.13335 PMC9113471 35313063

[mcn13562-bib-0005] Berry, N. J. , Jones, S. C. , & Iverson, D. (2012). Circumventing the WHO code? An observational study. Archives of Disease in Childhood, 97(4), 320–325. 10.1136/adc.2010.202051 21719442

[mcn13562-bib-0006] Cattaneo, A. , Pani, P. , Carletti, C. , Guidetti, M. , Mutti, V. , Guidetti, C. , & Knowles, A. (2015). Advertisements of follow‐on formula and their perception by pregnant women and mothers in Italy. Archives of Disease in Childhood, 100(4), 323–328. 10.1136/archdischild-2014-306996 25512963

[mcn13562-bib-0007] Cetthakrikul, N. , Kelly, M. , Banwell, C. , Baker, P. , & Smith, J. (2022). Regulation of baby food marketing in Thailand: A NetCode analysis. Public Health Nutrition, 25(10), 2680–2692. 10.1017/s1368980022001446 PMC9991832 35733357

[mcn13562-bib-0008] Chen, Y.‐C. , Chang, J.‐S. , & Gong, Y.‐T. (2015). A content analysis of infant and toddler food advertisements in Taiwanese popular pregnancy and early parenting magazines. Journal of Human Lactation, 31(3), 458–466. 10.1177/0890334415576513 25766374

[mcn13562-bib-0009] Ching, C. , Zambrano, P. , Nguyen, T. T. , Tharaney, M. , Zafimanjaka, M. G. , & Mathisen, R. (2021). Old tricks, new opportunities: How companies violate the international code of marketing of breast‐milk substitutes and undermine maternal and child health during the COVID‐19 pandemic. International Journal of Environmental Research and Public Health, 18(5), 2381. 10.3390/ijerph18052381 33804481 PMC7967752

[mcn13562-bib-0010] Chua, L. , & Win, A. Y. (2013). *Prevalence of breastfeeding in Singapore*. Vol. 4. https://www.singstat.gov.sg/-/media/files/publications/society/ssnsep13-pg10-14.pdf

[mcn13562-bib-0011] Doherty, T. , Pereira‐Kotze, C. J. , Luthuli, S. , Haskins, L. , Kingston, G. , Dlamini‐Nqeketo, S. , Tshitaudzi, G. , & Horwood, C. (2022). They push their products through me: Health professionals' perspectives on and exposure to marketing of commercial milk formula in Cape Town and Johannesburg, South Africa—A qualitative study. BMJ Open, 12, 055872. 10.1136/bmjopen-2021-055872 PMC9006797 35414555

[mcn13562-bib-0012] Grummer‐Strawn, L. M. , Holliday, F. , Jungo, K. T. , & Rollins, N. (2019). Sponsorship of national and regional professional paediatrics associations by companies that make breast‐milk substitutes: Evidence from a review of official websites. BMJ Open, 9(8), e029035. 10.1136/bmjopen-2019-029035 PMC6701639 31401600

[mcn13562-bib-0013] Han, S. , Chen, H. , Wu, Y. , & Pérez‐Escamilla, R. (2022). Content analysis of breast milk substitutes marketing on Chinese e‐commerce platforms. Maternal & Child Nutrition, 18(2), 13332. 10.1111/mcn.13332 PMC8932694 35213768

[mcn13562-bib-0014] Health Promotion Board . (2019). *The Sale of Infant Foods Ethics Committee Singapore (SIFECS) code of ethics*. https://www.hpb.gov.sg/docs/default-source/default-document-library/5th-edition-of-the-sifecs-code_as-of-020119.pdf?sfvrsn=8249c572_2

[mcn13562-bib-0015] Health Promotion Board Singapore . (2019). *Sale of Infant Foods Ethics Committee Singapore (SIFECS) code of ethics*. Health Promotion Board Singapore. Retrieved April 21, from https://hpb.gov.sg/healthy-living/food-beverage/sifecs

[mcn13562-bib-0016] Hernández‐Cordero, S. , Lozada‐Tequeanes, A. L. , Shamah‐Levy, T. , Lutter, C. , González de Cosío, T. , Saturno‐Hernández, P. , Rivera Dommarco, J. , & Grummer‐Strawn, L. (2019). Violations of the International Code of Marketing of Breast‐milk Substitutes in Mexico. Maternal & Child Nutrition, 15(1), 12682. 10.1111/mcn.12682 PMC7199041 30168899

[mcn13562-bib-0017] Hernández‐Cordero, S. , Vilar‐Compte, M. , Castañeda‐Márquez, A. C. , Rollins, N. , Kingston, G. , & Pérez‐Escamilla, R. (2022). Exposure to marketing of breastmilk substitutes in Mexican women: Sources and scope. International Breastfeeding Journal, 17, 16. 10.1186/s13006-022-00455-y 35236370 PMC8889386

[mcn13562-bib-0018] Hidayana, I. , Februhartanty, J. , & Parady, V. A. (2017). Violations of the International Code of Marketing of Breast‐milk Substitutes: Indonesia context. Public Health Nutrition, 20(1), 165–173. 10.1017/S1368980016001567 27323845 PMC10261563

[mcn13562-bib-0019] Jia Choo, P. , & Ryan, K. (2015). A qualitative study exploring first time mothers' experiences of breastfeeding in Singapore. Proceedings of Singapore Healthcare, 25(1), 5–12. 10.1177/2010105815615992

[mcn13562-bib-0020] Jones, A. , Bhaumik, S. , Morelli, G. , Zhao, J. , Hendry, M. , Grummer‐Strawn, L. , & Chad, N. (2022). Digital marketing of breast‐milk substitutes: A systematic scoping review. Current Nutrition Reports, 11(3), 416–430. 10.1007/s13668-022-00414-3 35507274 PMC9066994

[mcn13562-bib-0021] Karageuzián, G. , Vidal, L. , de León, C. , Girona, A. , & Ares, G. (2021). Marketing of commercial foods for infant and young children in Uruguay: Sugary products, health cues on packages and fun social products on facebook. Public Health Nutrition, 24(17), 5963–5975. 10.1017/s1368980021002780 34176550 PMC10195405

[mcn13562-bib-0022] Lau, Y. , Htun, T. P. , Lim, P. I. , Ho‐Lim, S. S. T. , Chi, C. , Tsai, C. , Ong, K. W. , & Klainin‐Yobas, P. (2017). Breastfeeding attitude, health‐related quality of life and maternal obesity among multi‐ethnic pregnant women: A multi‐group structural equation approach. International Journal of Nursing Studies, 67, 71–82. 10.1016/j.ijnurstu.2016.12.004 28012433

[mcn13562-bib-0023] Lisi, C. , de Freitas, C. , & Barros, H. (2021). The impact of formula industry marketing on breastfeeding rates in native and migrant mothers. Breastfeeding Medicine, 16(9), 725–733. 10.1089/bfm.2021.0041 33891498

[mcn13562-bib-0024] Liu, A. , Dai, Y. , Xie, X. , & Chen, L. (2014). Implementation of international code of marketing breast‐milk substitutes in China. Breastfeeding Medicine, 9(9), 467–472. 10.1089/bfm.2014.0053 25026262

[mcn13562-bib-0025] Lutter, C. K. (2013). The International Code of Marketing of breast‐milk substitutes: Lessons learned and implications for the regulation of marketing of foods and beverages to children. Public Health Nutrition, 16(10), 1879–1884. 10.1017/S1368980012004235 23034164 PMC10271411

[mcn13562-bib-0026] Merewood, A. , Grossman, X. , Cook, J. , Sadacharan, R. , Singleton, M. , Peters, K. , & Navidi, T. (2010). US hospitals violate WHO policy on the distribution of formula sample packs: Results of a national survey. Journal of Human Lactation, 26(4), 363–367. 10.1177/0890334410376947 20871089

[mcn13562-bib-0027] Nguyen, T. T. , Tran, H. T. T. , Cashin, J. , Nguyen, V. D. C. , Weissman, A. , Nguyen, T. T. , Kelly, B. , & Mathisen, R. (2021). Implementation of the code of Marketing of Breast‐Milk Substitutes in Vietnam: Marketing practices by the industry and perceptions of caregivers and health workers. Nutrients, 13(8), 2884. 10.3390/nu13082884 34445044 PMC8399411

[mcn13562-bib-0028] Parrilla‐Rodríguez, A. M. , & Gorrín‐Peralta, J. J. (2008). Formula labeling violations to the WHO code: A quantitative and qualitative analysis. Puerto Rico Health Sciences Journal, 27(1), 49–54. https://pubmed.ncbi.nlm.nih.gov/18450233/ 18450233

[mcn13562-bib-0029] Pereira‐Kotze, C. , Horwood, C. , Haskins, L. , Kingston, G. , Luthuli, S. , & Doherty, T. (2022). Exploring women's exposure to marketing of commercial formula products: A qualitative marketing study from two sites in South Africa. Global Health Action, 15(1), 2074663. 10.1080/16549716.2022.2074663 35946213 PMC9377257

[mcn13562-bib-0030] Pérez‐Escamilla, R. , Martinez, J. L. , & Segura‐Pérez, S. (2016). Impact of the Baby‐friendly Hospital Initiative on breastfeeding and child health outcomes: A systematic review. Maternal & Child Nutrition, 12(3), 402–417. 10.1111/mcn.12294 26924775 PMC6860129

[mcn13562-bib-0031] Pérez‐Escamilla, R. , Tomori, C. , Hernández‐Cordero, S. , Baker, P. , Barros, A. J. D. , Bégin, F. , Chapman, D. J. , Grummer‐Strawn, L. M. , McCoy, D. , Menon, P. , Ribeiro Neves, P. A. , Piwoz, E. , Rollins, N. , Victora, C. G. , & Richter, L. (2023). Breastfeeding: Crucially important, but increasingly challenged in a market‐driven world. The Lancet, 401(10375), 472–485. 10.1016/S0140-6736(22)01932-8 36764313

[mcn13562-bib-0032] Piwoz, E. G. , & Huffman, S. L. (2015). The impact of marketing of breast‐milk substitutes on WHO‐recommended breastfeeding practices. Food and Nutrition Bulletin, 36(4), 373–386. 10.1177/0379572115602174 26314734

[mcn13562-bib-0033] Richter, A. P. C. , Duffy, E. W. , Smith Taillie, L. , Harris, J. L. , Pomeranz, J. L. , & Hall, M. G. (2022). The impact of toddler milk claims on beliefs and misperceptions: A randomized experiment with parents of young children. Journal of the Academy of Nutrition and Dietetics, 122(3), 533–540.e533. 10.1016/j.jand.2021.08.101 34391941 PMC8840993

[mcn13562-bib-0034] Robinson, H. , Buccini, G. , Curry, L. , & Perez‐Escamilla, R. (2019). The World Health Organization Code and exclusive breastfeeding in China, India, and Vietnam. Maternal & Child Nutrition, 15(1), e12685. 10.1111/mcn.12685 30194804 PMC7199093

[mcn13562-bib-0035] Rollins, N. , Piwoz, E. , Baker, P. , Kingston, G. , Mabaso, K. M. , McCoy, D. , Ribeiro Neves, P. A. , Pérez‐Escamilla, R. , Richter, L. , Russ, K. , Sen, G. , Tomori, C. , Victora, C. G. , Zambrano, P. , & Hastings, G. (2023). Marketing of commercial milk formula: A system to capture parents, communities, science, and policy. The Lancet, 401(10375), 486–502. 10.1016/S0140-6736(22)01931-6 36764314

[mcn13562-bib-0036] Rollins, N. C. , Bhandari, N. , Hajeebhoy, N. , Horton, S. , Lutter, C. K. , Martines, J. C. , Piwoz, E. G. , Richter, L. M. , & Victora, C. G. (2016). Why invest, and what it will take to improve breastfeeding practices? The Lancet, 387(10017), 491–504. 10.1016/s0140-6736(15)01044-2 26869576

[mcn13562-bib-0037] Rollins, N. C. , Bhandari, N. , Hajeebhoy, N. , Horton, S. , Lutter, C. K. , Martines, J. C. , Piwoz, E. G. , Richter, L. M. , & Victora, C. G. (2016). Why invest, and what it will take to improve breastfeeding practices? The Lancet, 387(10017), 491–504. 10.1016/S0140-6736(15)01044-2 26869576

[mcn13562-bib-0038] Romo‐Palafox, M. J. , Pomeranz, J. L. , & Harris, J. L. (2020). Infant formula and toddler milk marketing and caregiver's provision to young children. Maternal & Child Nutrition, 16(3), e12962. 10.1111/mcn.12962 32157807 PMC7296786

[mcn13562-bib-0039] Rosenberg, K. D. , Eastham, C. A. , Kasehagen, L. J. , & Sandoval, A. P. (2008). Marketing infant formula through hospitals: The impact of commercial hospital discharge packs on breastfeeding. American Journal of Public Health, 98(2), 290–295. 10.2105/ajph.2006.103218 18172152 PMC2376885

[mcn13562-bib-0040] Rothstein, J. D. , Winch, P. J. , Pachas, J. , Cabrera, L. Z. , Ochoa, M. , Gilman, R. H. , & Caulfield, L. E. (2021). Vulnerable families and costly formula: A qualitative exploration of infant formula purchasing among peri‐urban Peruvian households. International Breastfeeding Journal, 16(1), 11. 10.1186/s13006-021-00356-6 33468169 PMC7816440

[mcn13562-bib-0041] De Roza, J. G. , Fong, M. K. , Ang, B. L. , Sadon, R. B. , Koh, E. Y. L. , & Teo, S. S. H. (2019). Exclusive breastfeeding, breastfeeding self‐efficacy and perception of milk supply among mothers in Singapore: A longitudinal study. Midwifery, 79, 102532. 10.1016/j.midw.2019.102532 31526969

[mcn13562-bib-0042] Sadacharan, R. , Grossman, X. , Matlak, S. , & Merewood, A. (2013). Hospital discharge bags and breastfeeding at 6 months: Data from the infant feeding practices study II. Journal of Human Lactation, 30(1), 73–79. 10.1177/0890334413513653 24305594

[mcn13562-bib-0043] Sheikh, S. P. , Akter, S. M. , Anne, F. I. , Ireen, S. , Escobar‐Alegria, J. , Kappos, K. , Ash, D. , & Rasheed, S. (2022). Violations of International Code of breast‐milk substitutes (BMS) in commercial settings and media in Bangladesh. Maternal & Child Nutrition, 18(S3), e13351. 10.1111/mcn.13351 35313083 PMC9113473

[mcn13562-bib-0044] Shing, J. S. , Lok, K. Y. , Fong, D. Y. , Fan, H. S. , Chow, C. L. , & Tarrant, M. (2022). The influence of the baby‐friendly hospital initiative and maternity care practices on breastfeeding outcomes. Journal of Human Lactation, 38(4), 700–710. 10.1177/08903344221086975 35403491

[mcn13562-bib-0045] Sobel, H. L. , Iellamo, A. , Raya, R. R. , Padilla, A. A. , Olivé, J. M. , & Nyunt‐U, S. (2011). Is unimpeded marketing for breast milk substitutes responsible for the decline in breastfeeding in the Philippines? An exploratory survey and focus group analysis. Social Science & Medicine, 73(10), 1445–1448. 10.1016/j.socscimed.2011.08.029 21978633

[mcn13562-bib-0046] Sokol, E. , Clark, D. , & Aguayo, V. M. (2008). Protecting breastfeeding in West and Central Africa: Over 25 years of implementation of the International Code of Marketing of breastmilk substitutes. Food and Nutrition Bulletin, 29(3), 159–162. 10.1177/156482650802900301 18947028

[mcn13562-bib-0047] Starks, H. , & Brown Trinidad, S. (2007). Choose your method: A comparison of phenomenology, discourse analysis, and grounded theory. Qualitative Health Research, 17(10), 1372–1380. 10.1177/1049732307307031 18000076

[mcn13562-bib-0048] Su, L. L. , Chong, Y. S. , Chan, Y. H. , Chan, Y. S. , Fok, D. , Tun, K. T. , Ng, F. S. P. , & Rauff, M. (2007). Antenatal education and postnatal support strategies for improving rates of exclusive breast feeding: Randomised controlled trial. BMJ, 335(7620), 596. 10.1136/bmj.39279.656343.55 17670909 PMC1989016

[mcn13562-bib-0049] Sue Howe . (2023). *Social media statistics in Singapore (updated 2023)*. Meltwater. Retrieved April 25 from https://www.meltwater.com/en/blog/social-media-statistics-singapore

[mcn13562-bib-0050] The Sale of Infant Foods Ethics Committee of Singapore . (2020). *The Sale of Infant Foods Ethics Committee of Singapore (SIFECS) code of ethics (updated December 2020)*. https://www.hpb.gov.sg/docs/default-source/default-document-library/5th-edition-of-the-sifecs-code_dec-2020.pdf

[mcn13562-bib-0051] UNICEF . (2023). *Baby‐friendly hospital initiative, ten steps to successful breastfeeding*. UNICEF. Retrieved July 19, from: https://www.unicef.org/documents/baby-friendly-hospital-initiative

[mcn13562-bib-0052] Victora, C. G. , Bahl, R. , Barros, A. J. D. , França, G. V. A. , Horton, S. , Krasevec, J. , Murch, S. , Sankar, M. J. , Walker, N. , & Rollins, N. C. (2016). Breastfeeding in the 21st century: Epidemiology, mechanisms, and lifelong effect. The Lancet, 387(387), 475–490. https://pubmed.ncbi.nlm.nih.gov/26869575/ 10.1016/S0140-6736(15)01024-7 26869575

[mcn13562-bib-0053] Vinje, K. H. , Phan, L. T. H. , Nguyen, T. T. , Henjum, S. , Ribe, L. O. , & Mathisen, R. (2017). Media audit reveals inappropriate promotion of products under the scope of the International Code of Marketing of breast‐milk substitutes in South‐East Asia. Public Health Nutrition, 20(8), 1333–1342. 10.1017/s1368980016003591 28294089 PMC10261673

[mcn13562-bib-0054] World Health Assembly . (1981). International Code of Marketing of Breastmilk Substitutes. World Health Organization. https://apps.who.int/iris/handle/10665/156596

[mcn13562-bib-0055] World Health Organization . (2017a). Guidance on ending the appropriate promotion of foods for infants and young children: Implementation manual . https://apps.who.int/iris/bitstream/handle/10665/260137/9789241513470-eng.pdf

[mcn13562-bib-0056] World Health Organization . (2017b). Guideline: Protecting, promoting and supporting breastfeeding in facilities providing maternity and newborn services . https://www.who.int/publications/i/item/9789241550086 29565522

[mcn13562-bib-0057] World Health Organization . (2022a). Global breastfeeding scorecard 2022: Protecting breastfeeding through further investments and policy actions . https://www.who.int/publications/i/item/WHO-HEP-NFS-22.6

[mcn13562-bib-0058] World Health Organization . (2022b). *How the marketing of formula milk influences our decisions on infant feeding* (978‐92‐4‐004460‐9). https://www.who.int/publications/i/item/9789240044609

[mcn13562-bib-0059] World Health Organization . (2022c). *Marketing of breast‐milk substitutes: National implementation of the international code, status report 2022*. https://www.who.int/publications/i/item/9789240048799

[mcn13562-bib-0060] World Health Organization, & UNICEF . (2003). *Global strategy for infant and young child feeding*. https://apps.who.int/iris/bitstream/handle/10665/42590/9241562218.pdf

[mcn13562-bib-0061] Wright, C. M. (2006). Relationships between paediatricians and infant formula milk companies. Archives of Disease in Childhood, 91(5), 383–385. 10.1136/adc.2005.072892 16632663 PMC2082719

