# Supplementary material for: Commercial milk formula marketing following increased restrictions in Singapore: A qualitative study
Source: Matern Child Nutr. 2023 Sep 5;20(1):e13562. doi: 10.1111/mcn.13562 (PMC10750007; doi:10.1111/mcn.13562)
Supplement: Supplementary file 1 — Supporting information. [file MCN-20-e13562-s001.docx]

| **Theme** | **Sub-theme** | **Sample quotation(s)** |
| --- | --- | --- |
| **Marketing to the general public** | | |
| As experienced by mothers | Online advertising | “I do see them [formula adverts] pop up on Facebook, I think, because of what I googled right.” Mother (Breastfeeding)  “Instagram ads. So when you are looking at stories and all, they will just pop up. But they were just promoting in general… normally they will only promote the stage 3.” Mother (formula feeding)  “I also signed up for the Nestlé Mom's pack… It just kept appearing in my feed.” Mother (formula feeding) |
|  | Follow-up engagement | “I signed up for some of the samples... So after that, they would routinely call me to offer samples for the next stage.” Mother (Breastfeeding)  “…the moment you put in any data, they will call you.” Mother (Breastfeeding) |
|  | Samples and coupons | “So they give you the samples and they come with discount codes.” Mother (Breastfeeding)  “I remember when my child was almost two and we switched to Aptamil because they gave you vouchers, and it is cheaper… That's how they made their market in Singapore. So Aptamil is now very popular.” Mother (Breastfeeding) |
| As observed by health workers | Free gifts | “The freebies that they give are very attractive. They will say, ‘Buy $100 or $200 worth of this formula, you will get this free.’ Their gifts are expensive ones, maybe a play gym or whatever.” Lactation consultant |
|  | Education and research | “There are a lot of subtle education on how formula is so advanced nowadays, with all the research and all that. Then it gives parents false information, like ‘actually formulas are pretty good and is really state of the art.’” Paediatrician |
|  | Premium nutrition claims | “These competitors try to price their products at a premium to say that because the formulation they have has X number of nutrients better than the other competitor, compared to their rivals or this is the only one with whatever added.” Doctor (women’s health)  “Well, they are very pricey. They're very good at targeting what mothers think about, like they say about the different vitamins, the different growth stages, all the extra proteins it has.” Nurse |
|  | Line extensions | “Why they have moms' milk is that the effect of, ‘Ha. I'm drinking this milk, so my baby must drink the same brand of the infant formula.’ They have all these kinds of marketing tactics and strategies.” Paediatrician  “They actually start you from when you are pregnant, maternal milk… Then they also move on to toddler milk… You know they have this special formula for picky eaters.” Lactation consultant |
| **Marketing in the healthcare setting** | | |
| Marketing targeting mothers | Gifting of branded goods | “Oh you have Abbott, Abbott and their growth charts… paediatricians, they do have all those sponsored… wallpaper and stuff like that.” Mother (Breastfeeding)  “You can see some of the toys that he [paediatrician] places there for the kids to play. You can see the brand names.” Mother (Breastfeeding) |
|  | Promotion at hospital pharmacy | “…you go to the [hospital] pharmacy, then you see formula milk there.” Mother (Breastfeeding)  “I passed by one of the pharmacies. Over there, they did have a standee or a booth for Aptamil... they did give some talk and they also offered free samples for their Stage 4 product.” Mother (formula feeding) |
|  | Free samples | “I think I have seen Nan, the Nestlé brand, and then I seem to have seen Abbott, Similac, and Enfagrow as well. They have a sample sachet for you know, when I see a paediatrician, they just give us some [toddler milk] samples.” Mother (Breastfeeding)  “When I was pregnant and when I was doing my routine gynae visit, they also gave us the sample… the Nestlé Mom powder... Not the infant one, just for the lactation.” Mother (formula feeding)  [In paediatric clinic:] “They put all the milk samples by all the brands out there for you to choose… It's really for you to take as much as you like.” Mother (Breastfeeding) |
|  | Promotional items in discharge bags | “There were a few vouchers for photoshoots and… a few products you can buy like Similac Mom.” Mother (formula feeding)  “[In the private hospital] there are two kinds of discharge bags. One is prepared by [the hospital], another one, I think is another bag that is by a formula brand… you will see Friso giving you bottles, containers, all that, with their names… Similac also gave out some bibs.” Mother (Breastfeeding) |
|  | No promotion of infant formula | “Even if you contact them when the kid is born, they wouldn't offer you any samples until after six months. So they cannot offer any samples for stage one I think, it's against the local guidelines.” Mother (Breastfeeding)  “I got the goodies pack, but they didn't give any baby formula sample, not at all.” Mother (formula feeding) |
|  | Rotation of formula brands | “It was Dumex at that time. I heard that they changed the brand… they can't promote a certain brand so they change it… Every month there seems to be a different brand.” Mother (formula feeding) |
| Marketing targeting health workers | Gifting of branded goods | [On seeing a calendar with infant formula company logo in polyclinic:] “It's labelled there that it is only for health professionals’ use, but I spotted it.” Nurse |
|  | Free samples of CMF | “[On milk for mothers:] it's not really medically needed, so most of us don't actually promote it to our patients. Although that one they do try to advertise to us.” Doctor (women’s health)  “They say, ‘Would you like to take some of this milk for the mother?’ They had done their own study that it helps the mother, those who are not eating well. Sometimes we do take some mother's milk… We put it out there, indirectly in a way, telling moms, ‘If on that day you're too busy, you're unable to eat well…then you have a drink because it's a pack of goodness.’” Lactation consultant |
|  | Seeking professionals’ endorsement | “I've seen samples of it [Enfamil ‘Mums-To-Be’ milk] before… They [reps] asked me, ‘Ask your patients [to] buy.’” Doctor (women’s health)  “They are allowed to approach us and provide us with scientific evidence, sharing their products. I've met them… From them, I actually get more information about their products, quite interesting what they believe is the difference between the different ranges. I ask them the cost of it and they're quite helpful.” Paediatrician |
| Seeking partnership with health workers | Unaware of any collaboration | “Personally, I've not heard about any collaboration research or any seminars that we allow for people from the formula milk industry.” Doctor (women’s health)  “now they don't dare to try anymore because it'll be a flat ‘no’.” Lactation consultant |
|  | Collaboration is restricted but still exist | “I think [another doctor] was doing something with one of the companies… she had a real trouble recruiting because of the BFHI [Baby Friendly Hospital Initiative] policy.” Doctor (women’s health)  “…they don't pedal milk to you, but they will want to collaborate with the College of [Paediatricians], with professional societies to say, ‘Hey, let's sponsor some talks. We can organize this and that.’ I think those are subtle ways that they are still trying to maintain the relationship.” Paediatrician |
|  | Education initiatives | “…we have Journal Club, then they tried to sponsor the food for Journal Club and the [industry] guys are educating us about new developments in formula milk.” Doctor (women’s health)  “They do set up a lot of these so-called educational arms that reach out to not only moms, but physicians as well. Nestle Nutrition Institute, as an example… there are a lot of subtle education on how formula is so advanced nowadays, with all the research and all that... Even for physicians as well, then they get the misconception that there's nothing wrong with formula-feeding, it's as good as breastfeeding.” Paediatrician  “On occasion that they actually are able to bring experts and they come and give us educational talk, CME [Continuing Medical Education] talk, then we accept, but without food provided, freebies provided.” Paediatrician |
|  | Overseas conferences | “They still do sponsor doctors to go for overseas conferences and so forth, unfortunately.” Paediatrician |
|  | Research collaboration | “They also got involved with nutrition research and invested a lot in this, getting into the good books of doctors and scientists.” Lactation consultant |
| **Health workers’ attitudes towards marketing and its restriction** | | |
| Views on the industry and its marketing | Profit-based | “Formula companies, they are professional organizations, profit-making.” Paediatrician  “I think it is even more well developed than the breastfeeding groups that we have because clearly, [infant formula] is an important source of revenue [for the companies].” Doctor (women’s health) |
|  | Well-resourced | “So, in terms of branding, whichever brand or company has more money, in a sense they are able to have the luxury to market.” General practitioner  “The industry is huge…one particular [company] whose annual [global] turnover is 35% of Singapore's GDP.” Paediatrician |
|  | Sufficiently regulated | “They seem to know their parts [place]… they never push me to offer anything.” Lactation consultant  “Actually, the hospital is quite strict now, so definitely you're not allowed to promote formula milk.” Doctor (women’s health) |
|  | Not necessarily a problem | “I think you're not allowed to carry even formula milk [in the hospital pharmacy]…I personally think that is a little bit extreme, especially for the first week. Like I said, my personal mantra is milk is better than no milk at all.” Doctor (women’s health) “[Marketing] for mother's milk, I think, it's no harm. It's a form of beverage. I think that should be okay. You're helping moms if they're unable to eat well…like essence of chicken.” Lactation consultant |
|  | Still a problem | “Even for physicians as well that they get the misconception that there's nothing wrong with formula-feeding, it's as good as breastfeeding. We have spoken to moms as well who actually think that formula is superior to breast milk.”- Paediatrician  “Every mom and parent [in Singapore] tend to think that the child needs formula, even post one year and all of that… I don't know why. Is it because of marketing, or is it because of something else?” Researcher |
|  | Needs to be regulated | “It still has to be governed by the ethical principles of supporting breastfeeding, and I think it should still be subject to regulations.” Paediatrician |
|  | Sophisticated | “They are professional organizations, profit-making. They are always one step ahead of us.” Paediatrician  “I think that formula milk [industry], their advertising is very good.” Lactation consultant |
| Views on industry partnership | Has practical value | “…funding for research is competitive and you have to be realistic… They have an influence on the world. They're also stakeholders… When their companies were founded, they were solving problems. Things changed a lot, of course, so we have to all pivot to the new norms.” Paediatrician |
|  | Approach with caution | “It's like with the drug companies, it's always a fine line, because you want to know about the drugs, but you don't want them to influence your decisions.” Doctor (women’s health)  “The end agreement will need to be very clear and you need to be very careful.” Researcher |
|  | To be avoided altogether | “The research is always a bit discounted, because definitely, we can't exclude some covert influence. Sometimes you may not even realize that you've been influenced.” Doctor (women’s health)  “Professional activities should be independent of commercial sponsorships because it's really a conflict of interest and it dilutes also, sometimes, our aspirations and our professionalism.” Paediatrician  “I would be reluctant to collaborate with the industry because based on my previous experience in collaborating with them, somehow they would put in some enforcement [constraints] over you. For example, some of the findings may not able to be published, and then every single findings you would need to go through with them in very detail.” Researcher |
| Awareness of and adherence to restrictions | Unaware of any policies | “I’m not aware of any written policies. They may be there. Just that I don't know about them.” Doctor (women’s health) |
|  | No policy but discouraged | “I don't think we have a blanket policy, per se, but I don't think we're encouraged to do research or collaborate with the formula milk industry… given that we are 100% breastfeeding hospital.” Doctor (women’s health) |
|  | Ethics policy for research | “All our research goes through the ethical process, and of course, we abide by the Helsinki rules. It's all governed.” Paediatrician  “We will have to agree to sign off the CRA [Collaboration Research Agreement]. From there, there are a lot of terms and conditions, especially they are not allowed to interrupt any analysis or conclusions or findings, these kinds of things.” Researcher |
|  | No promotions in hospitals | “There's a breastfeeding policy. In that, we are adhering to-- It's not just our hospital, it’s all hospitals in Singapore. It's under SIFECS. You cannot get sponsorship or free gifts from the formula company.” Paediatrician  “So they will try to sneak their way in, yes. Then give free talk to the nurses and all, then we'll just block them all off. They give free gifts; we won't be able to accept any of it.” Lactation consultant |
|  | No brand endorsement | “We do not endorse any brands, we always tell the parents, all formula feeds, all good.” General practitioner  “We are bound by the code, so we cannot give free formula. That means that we will charge for every formula feeding. We will not recommend any brands.” Lactation consultant |
|  | Gatekeeper system | “I would say that, at least I can speak for my hospital, anything to deal with formula, they come to me. Even our corporate communications, they know and they're very mindful.” Paediatrician |
|  | Variations between public and private health settings | “…in the private hospitals, as far as I know, that's where most of these formula milk companies make their money. I think they're not as strict [as public hospitals], and they're clearly more pro-choice, they run it more like a service up there, compared to an institution.” Doctor (women’s health)  “I think for the three public hospitals, the obstetricians are very involved. I would say that there is quite a lot of emphasis on supporting and discussing about breastfeeding. For private hospitals, because O&G physicians are basically just practitioners, they rent the clinic, each of them have their own practice.” Paediatrician |
| **Health workers’ attitudes and practices on infant feeding** | | |
| Views on formula feeding | Appropriate if medically indicated | “Okay. There are some conditions which are contraindicated for breastfeeding like HIV, certain infectious diseases, there's a list of these. So for these we would say not to breastfeed. The other thing would be, so if the baby is not gaining weight well and things like that, usually we don't have to stop, you can supplement but you try to protect the supply so that they can still resume if they need to.” Doctor (women’s health)  “Most of the times is medical indications, whichever way you look at it because it's medical indications for maternal illness, child's needs, insufficient milk.” Paediatrician |
|  | Appropriate if breastfeeding difficult | “There are so many possible difficulties in the process of breastfeeding, from maternal health, maternal mental health, maternal nipple health, maternal breast congestion. There's so many things, you've got to think about all the individual things. There will be a group of women who cannot sufficiently breastfeed, and they shouldn't be looked negatively upon because these are real things… Then the third one would be for mothers who for some reason… cannot be near the babies all the time. They need access to milk formula otherwise babies won't thrive.” Paediatrician |
|  | Appropriate as supplement | “As we said again, patients who do not produce enough milk initially, some of them may carry on and not produce enough milk ever. But you don't expect the baby to starve, so give the baby the formula.” Doctor (women’s health)  “If the mother is actually too stressed… I will say to her, ‘Do what you're doing now. You've done a good job already. Try to maintain that amount. Then we top up for the other part of it.’ That is better than none. Rather than stressing her so much and then she packs up and gives up breastfeeding.” Lactation consultant |
|  | More nutritious | “If I'm not wrong, there are some pieces of evidence that say that formula milk is actually more nutritious than breastmilk after the first six months.” Doctor (women’s health) |
|  | Problematic | “It used to be a solution, and now it's become a problem. In our world, half a century ago, we needed infant formula because the mothers were undernourished.” Paediatrician  “For babies right, once you introduce their formula milk, formula milk is sweet right, and then they don't want to come back to the breastmilk…suckling needs a lot of energy.” Researcher |
|  | Should be medication | “I think that's what we are aiming for, only special kids… they have to have a specially designed formula, then they will have to be given by their paediatrician. They shouldn't actually be available for selling.” General practitioner |
| Factors influencing feeding advice | Mother’s underlying reasons | “Usually, I would try to explore what's the underlying reason…sometimes it's due to misconceptions. Then in those cases, it'd be useful to clear these misconceptions.” Doctor (women’s health) |
|  | Respecting patient choice | “I think it's still a personal choice, but I believe that it is important for them to make an informed decision and at the same time, not to make them feel like they are any less of a parent just because they choose to give formula.” - Paediatrician  “Ultimately I think it's still a mother's right to choose whether she wants to breastfeed or formula feed.” Nurse |
|  | Personal interest | “I think it's basically whether you got an interest in it or you don't. I suppose if you are very interested in the subject, you're very passionate about the subject, I think it helps to convey that same enthusiasm to the patient.” - Doctor (women’s health)  “Some doctors, maybe they are not interested in breastfeeding because in medical school, you only got one lecture on breastfeeding… some schools of thought, they are not supportive of breastfeeding.” - Lactation consultant |
|  | Age | “I feel it's generational because for the younger doctors… breastfeeding is the thing that we promote. Older doctors come from a time where breastfeeding was thought of as not so important.” - Doctor (women’s health) |
|  | Gender | “I must say there is probably a slight difference between the male obstetrician and the female obstetrician. Generally, the males are ‘okay, whatever.’ that sort of thing. While the females are probably a little bit more encouraging about it.” - Doctor (women’s health) |
| Mothers’ experiences with feeding advice | Breastfeeding strongly encouraged | “but it was like the norm for all of them, that everyone had to, all mothers will just breastfeed. So they didn't really even ask, they just bring the baby to you, bring the baby to your nipple and then start to feed.” Mother (formula feeding)  “When we are registering for our [prenatal] scans or the gynae visits, they will give you the pamphlet [on breastfeeding]… In fact, when I was waiting for my scan once, there was a lady who walks around… to encourage you to go for lessons.” Mother (Breastfeeding) |
|  | Breastfeeding an option | [Child had jaundice:] “They were saying ‘would you want to give formula so it goes down faster.’ So it was an option, but she was telling me that maybe you should pump. So I think it was quite balanced.” Mother (Breastfeeding)  “Before I gave birth they already asked, ‘Do you intend to breastfeed, or do you intend to feed formula?’… They make it sound as if breastfeeding is optional.” Mother (Breastfeeding) |
|  | Staff offered formula | “The first hour they gave her formula milk because she was low on sugar because there was no breastmilk at that time… because I was sedated.” Mother (Breastfeeding)  [Post-caesarean:] “They didn't ask us whether we want the donated breast milk from the milk bank, or we want formula. I don't think there was a choice.” Mother (formula feeding) |
| **Mothers’ attitudes towards marketing and formula feeding** | | |
| Mothers’ formula brand choices | Expert endorsement | “Some were bloggers and some were, I think they were lactation consultants from other countries and they were just recommending that certain brands taste quite similar.” Mother (Breastfeeding)  “My baby had some colic issues... PD [paediatrician] recommend[ed] Similac… That's why we decided to change to Similac… I'm not sure why the PD chose that brand.” Mother (formula feeding) |
|  | High quality conveyed by price | “I should just pick the most expensive one, because I already could not give my kid enough breast milk, so I thought I'd better pick the most expensive one, assuming that that would be the best.” Mother (Breastfeeding)  “If I had the financial capacity, of course, I would go for the branded ones, the really expensive ones, assuming that the more expensive it is, the more nutritional it is.” Mother (Breastfeeding) |
|  | Value for money | “I'm using Nature One now because it is very cheap.” Mother (formula feeding) |
|  | Nutritional components | “…they were saying the gold bottle is better because it has probiotics.” Mother (Breastfeeding) |
|  | Trusted brand | “What is the company? Is it Abbott? Is it Nestlé? I would see whether it is something that is established, basically, so that at least it's not so scary.” Mother (formula feeding) |
|  | No major difference | “So far, we can't see any difference between the formulas.” Mother (Breastfeeding)  “It is cow's milk powder plus and minus some certain ingredients. I think it is just branding.” Mother (formula feeding) |
| Views on marketing | Relatively innocuous | “There's a lot of hoo-haa over the formula companies advertising but to be honest, for me, I don't think it's a huge factor... everyone does know that breast milk is best. It's not because they've been brainwashed by the formula companies. It's just personal constraints that make breastfeeding very difficult.” Mother (Breastfeeding) |
|  | Supports breastfeeding | [On why she felt Nestlé supports breastfeeding:] “I think generally when they sponsor stuff, they don't have anything that talks about formula milk.” Mother (Breastfeeding) |
|  | Dubious nutritional claims | “Pediasure is a big thing because they always talk about picky eaters. Picky eaters, if you give them formula milk, that's not going to help with picky eating.” Mother (Breastfeeding)  “A lot of these claims, you don't know how much you can trust.” Mother (Breastfeeding) |
| Views on formula feeding | Unnecessary | “To me, I don't feel like you really need to feed formula.” Mother (Breastfeeding) |
|  | Has unhealthy additives | “One thing I don't like formula also is the sugar content… it's a lot of additives.” Mother (Breastfeeding) |
|  | No different to breastmilk | “In terms of nutritional benefits, I would say at this point I'm not so sure. I'm not an expert, but I think it's quite negligible because it's just food.” Mother (Breastfeeding)  “For me, formula milk is still the same. Baby is still drinking milk… if the formula is not safe, our government won't allow shops to be selling it.” Mother (formula feeding) |
|  | Being fed is most important | “I think some parents tend to overdo it to the extent that they think that formula is poison. Which is also quite bad because sometimes it comes to a point whereby they neglect whether their child is fully fed or not.” Mother (Breastfeeding)  “most… important is that our baby is fed in general, and it should not be too stressful to choose either one because we also do not want to make our life more difficult.” Mother (formula feeding) |
|  | Matter of personal choice or situation | “I am managing to do it but I think it would be unfair to expect anyone to do something similar.” Mother (Breastfeeding)  “Some people, they choose to 100% feed their child formula and the child also grow up healthy and happy. I don't think it is a negative thing, it is just the decision of the parents how to feed your child.” Mother (formula feeding) |
