# Supplementary material for: Commercial milk formula marketing following increased restrictions in Singapore: A qualitative study
Source: Matern Child Nutr. 2023 Sep 5;20(1):e13562. doi: 10.1111/mcn.13562 (PMC10750007; doi:10.1111/mcn.13562)
Supplement: Supplementary file 2 — Supporting information. [file MCN-20-e13562-s002.docx]

**Supplementary file 1: Interview Guides for Mothers and health-workers**

| **I. Mothers** |
| --- |
| Background information   1. Please tell me a bit about your child and current childcare arrangement. 2. Please tell me a bit about your breastfeeding journey. 3. What healthcare services in Singapore did you use in relation to the delivery and care of your child?   Infant feeding   1. Did any of your healthcare providers discuss with you about infant feeding options? 2. What kind of breastfeeding support did you receive during and after your delivery? 3. Did you receive a hospital discharge pack? What were the contents of the pack? 4. What are your views on breastfeeding and formula feeding? 5. If mothers are formula-fed: which brand of formula do you use? How did you decide on this brand over those brands?   CMF industry   1. Have you ever been approached by representatives of formula companies? What was your experience like? 2. Have you ever been offered formula samples or promotional/discount coupons? 3. Can you recall where you have seen formula brands, logos, and promotions? |
| **II. Health-workers** |
| Feeding support and practice   1. Please briefly describe your role within your organization. 2. In what ways does the topic of infant feeding come up during your care journey with mothers? 3. What is your approach for women who opt not to breastfeed without any medical indications? 4. What is your hospital or practice’s policy or standard process to encourage breastfeeding? 5. Under what circumstances are mothers advised to stop breastfeeding? 6. What is your hospital’s approach if a mothers asks for her baby to be fed formula, without any medical indications?   Formula feeding   1. Does your hospital have a policy or standard process on when to give pre-lacteal or supplemental feed to infants? Please describe the policy. 2. In your hospital, typically, what is the responses to requests by parents to feed their baby formula without medical indications? 3. For mothers who opt to give pre-lacteal or supplemental feed, does your hospital continue to offer formula to the mother, or check her preference for each feed? 4. What is advice given to mothers regarding formula-feeding? 5. What is your views on formula feeding? 6. What are the reasons given by mothers who have opted to formula-feed partially or entirely?   CMF industry   1. Do representatives from formula companies approach you or your clinic/hospital? Can you please share your experience? 2. What types of collaboration does your hospital have with companies that manufacture or sell formula? Is it similar across all departments? 3. Have formula companies offered gifts or sponsorships to your hospital (e.g. talks for staff, conference sponsorships, etc.)? 4. What are the types of items included in your hospital discharge bag? 5. Does your hospital have a policy on interactions with commercial entities such as formula companies? How about your department? 6. What are your views on the formula industry in Singapore? 7. What are your views on collaborations between the healthcare industry and formula manufacturers? 8. What are your views on WHO’s code on formula marketing? 9. Are you familiar with the BFHI? Where did you hear about it? 10. What are your views on how well BFHI has been implemented in Singapore? |
